# Building digital patient pathways for the management and treatment of multiple sclerosis

**DOI:** 10.3389/fimmu.2024.1356436

**Published:** 2024-02-15

**Authors:** Judith Wenk, Isabel Voigt, Hernan Inojosa, Hannes Schlieter, Tjalf Ziemssen

**Affiliations:** ^1^ Center of Clinical Neuroscience, Department of Neurology, Faculty of Medicine and University Hospital Carl Gustav Carus, Technische Universität Dresden, Dresden, Germany; ^2^ Research Group Digital Health, Faculty of Business and Economics, Technische Universität Dresden, Dresden, Germany

**Keywords:** multiple sclerosis, patient pathway, clinical pathway, digital pathway, artificial intelligence, digital health, connected health, digital twin

## Abstract

Recent advances in the field of artificial intelligence (AI) could yield new insights into the potential causes of multiple sclerosis (MS) and factors influencing its course as the use of AI opens new possibilities regarding the interpretation and use of big data from not only a cross-sectional, but also a longitudinal perspective. For each patient with MS, there is a vast amount of multimodal data being accumulated over time. But for the application of AI and related technologies, these data need to be available in a machine-readable format and need to be collected in a standardized and structured manner. Through the use of mobile electronic devices and the internet it has also become possible to provide healthcare services from remote and collect information on a patient’s state of health outside of regular check-ups on site. Against this background, we argue that the concept of pathways in healthcare now could be applied to structure the collection of information across multiple devices and stakeholders in the virtual sphere, enabling us to exploit the full potential of AI technology by e.g., building digital twins. By going digital and using pathways, we can virtually link patients and their caregivers. Stakeholders then could rely on digital pathways for evidence-based guidance in the sequence of procedures and selection of therapy options based on advanced analytics supported by AI as well as for communication and education purposes. As far as we aware of, however, pathway modelling with respect to MS management and treatment has not been thoroughly investigated yet and still needs to be discussed. In this paper, we thus present our ideas for a modular-integrative framework for the development of digital patient pathways for MS treatment.

## 1 Introduction

Many roads lead to Rome, or so the saying goes. The same is true for multiple sclerosis (MS). There is no such thing as one unique disease course in MS. MS is one of the most common disabling neurological diseases in young adults (1, 2). Focal and diffuse neuroinflammation combined with neurodegeneration in the central nervous system translates into a chronic disease which evolves very differently over time, with people experiencing an array of diverse symptoms or functional impairment in form of relapses and gradual disability progression (3). A cure for MS has not been found yet, but, in particular in the last decade, a growing number of MS-specific disease modifying therapies have become available which can be applied to modify the pathophysiological processes and to thus slow and mitigate its progression (4).

For all of these reasons, there is no straight-forward way to treat MS. In effect, MS course is characterized by a high intra- and inter-individual variability (5–7). For achieving best-possible outcomes in a patient, health care professionals (HCPs) need to carefully consider the specific disease course of the patient in front of them. Hence, MS treatment needs to be tailored to the individual patient’s needs and requirements to take full effect. MS treatment and management are thus highly complicated and complex. People with MS (pwMS) and their doctors, nurses and caregivers alike would thus presumably greatly benefit from a tool helping them to navigate MS treatment and to coordinate their efforts to keep the disease in check.

We are convinced that digital patient pathways – if properly designed and applied – are the best tool available for this purpose. Recent advances in the field of artificial intelligence (AI) open new possibilities regarding the interpretation and use of big data from not only a cross-sectional, but also a longitudinal perspective. This is also why digital pathways are essential for putting modern MS management into practice, i.e., by building and introducing digital MS twins to MS care (8, 9). However, as far as we are aware, the potential use of digital pathways in MS management and approaches to the implementation of pathways systems in MS care have not been further investigated yet. In this paper, we thus set out to close this research gap and present a modular-integrative framework for the further development of digital patient pathways for MS treatment.

The concept of pathways in medicine goes a long way back. Therefore, we will first go on to elaborate on the history, characteristics and functions of pathways in healthcare in general in, before explaining our vision of digital patient pathways for MS in more detail.

In conclusion, we will explain how digital patient pathways in combination with AI may also lead to the discovery of new MS phenotypes and why digital patients, metaphorically speaking, may be considered the arteries of any kind of digital twin for MS. In the fourth section then, we will touch upon major challenges that are likely to arise when it comes to building digital patient pathways in the real world. Finally, we will provide a short summary of all sections and an outlook on future MS research.

## 2 Past and present: pathways and their application in healthcare

**Figure 1:**
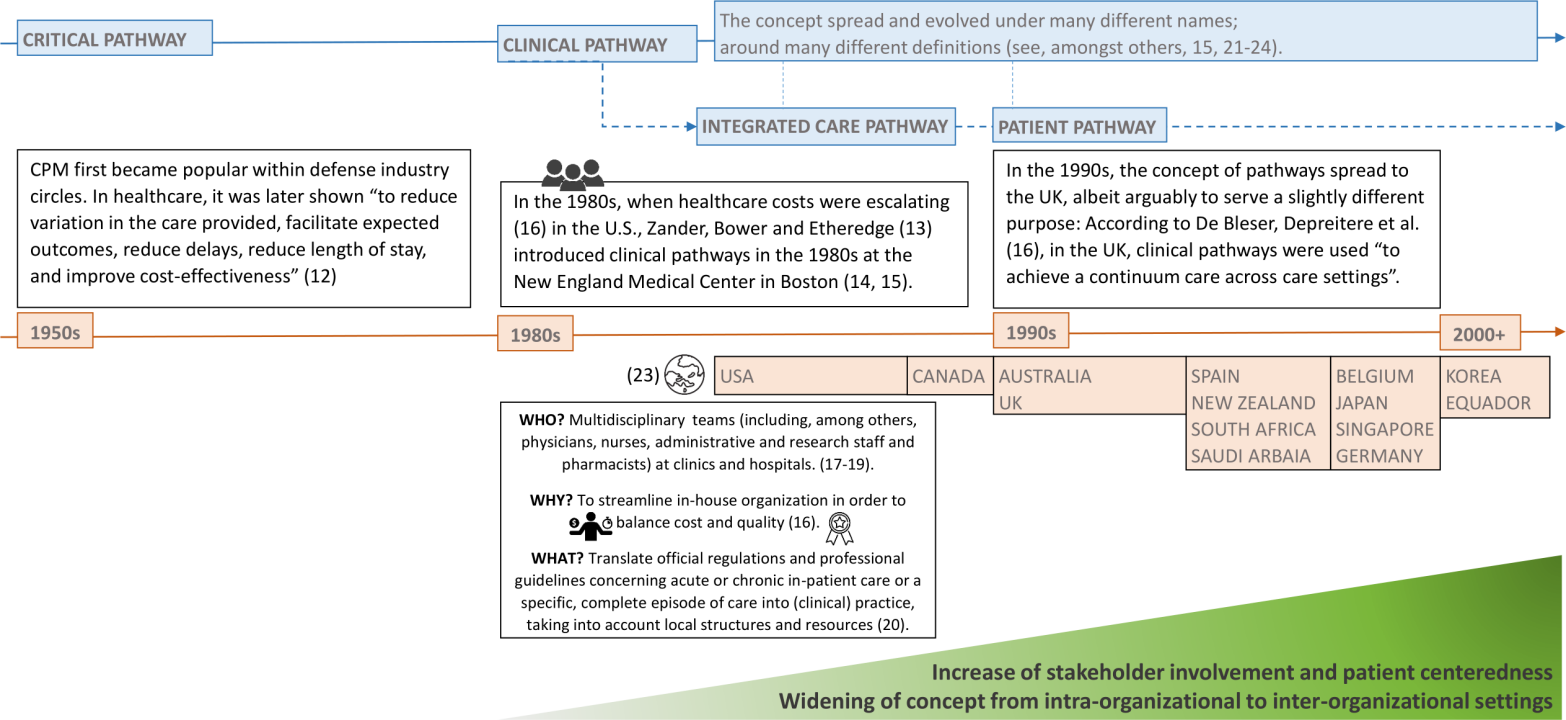
The conceptual evolvement of pathways in healthcare over time. Source: Self-prepared, icons created by Freepik – Flaticon, https://www.flaticon.com/free-icons/.

Ever since money became an issue in healthcare, process optimization and quality management have attracted growing attention among healthcare researchers, practitioners and policy makers (10). In search of solutions, they started to bring management techniques such as the Critical Path Method (CPM), Six Sigma, or the Just-In-Time concept by Toyota into healthcare (11). This eventually led to the introduction of clinical pathways in the 1980s. Since then, the concept of pathways has spread around the world and has steadily evolved in different directions and under various names [see **Figure 1** based on (12–24)] which include, e.g., the following to name just a few: *critical pathway*, *clinical pathway*, *integrated care pathway*, *care pathway* and *care map* (16).

Kinsman, Rotter et al. (24) set out to resolve the conceptual confusion and developed an operational definition for what could actually be deemed a *clinical pathway*, independent of the exact terminology used. They came up with five criteria and tested their usefulness against 263 publications (22, 23). In their review they also included studies which did not explicitly use the term *clinical pathway*, but used terms such as “care model”, “care map” or even plainly “protocol” instead. More recently, Richter and Schlieter conducted a scoping review (25) in relation to the term *patient pathway* as well as a later survey (26) to validate their concept of patient pathways.

Both pathway concepts are presented in **Table 1**, highlighting overlapping contents and differences, which mirror the conceptual evolvement over time. As shown there, patient pathways constitute an extended version of clinical pathways: They include more stakeholders at all stages of their life cycle (development, implementation and use) and shift the focus to the patient. This was not common in the past, but has been increasingly advocated by researchers (18–20).

**Table 1:** From clinical pathways to patient pathways.

According to Kinsman, Rotter et al. (24), a ***clinical pathway*** can be defined to be…	Whereas a ***patient pathway***, according to Richter and Schlieter (25, 26) …	
(1) … *a structured multidisciplinary plan of care* which meets at least three of the remaining four criteria.	…. prescribes the *timely sequence of key activities* that are to be performed *in a process of care* which overall make up a *patient’s journey* through the health system.	It therefore *extends* the step-by-step description of the care process *from intra-organizational settings to* cover the whole care chain across in- and outpatient care, i.e., *inter-organizational settings*, and …
(2) It allows for the translation of guidelines or evidence into local structures,	It describes the *functional, biological and patient-related goals and milestones of care* for individual patients of a specific, homogenous patient population with complex chronological conditions. It is used for *patient information, documentation, monitoring* and the *evaluation* of the care process in terms of, e.g., quality, efficiency, PROMs.	
(3) it details the steps to be taken in a course of treatment or care in a plan, pathway, algorithm, guideline, protocol or other “inventory of actions”,		
(4) it supports the standardization of care for a specific clinical problem, procedure, or episode of healthcare in a specific population (22, 24).	It is *developed, implemented and used* by a *multidisciplinary care team* consisting of HCPs, professional and informal care givers *as well as the patient*. Thereby it serves as an *evidenced-based navigation tool* for patients and all professional and informal caregivers involved in the care process.	… puts strong emphasis on patients’ needs and preferences. Thereby it also supports *patient empowerment* and *patient engagement*.

Source: Self-prepared.

Karen Zander, one of the early pioneers promoting the implementation of clinical pathways in the 1980s, however, already back then prompted the evolution of pathways in this direction. According to De Bleser, Depreitere et al. (16), in her article of 1988 (27), she also suggested to widen the concept of pathways to incorporate *all* aspects of patient care, i.e., including prospective plans for all disciplines involved in patient care. In this regard, Zander then started to speak of *integrated care pathways* [see, e.g., (21)].

With regard to chronic diseases such as MS, and the variety of different HCPs being involved in MS care due to the disease’s overall complexity, this conceptual development deserves special attention. Up until now, there are only few articles available on pathways concerning MS care, even though pathways in healthcare in general received a lot of attention in research and in practice in the past. Examples in the area of MS include integrated care pathways (paper-based) for MS rehabilitation (28), a clinical pathway (paper-based, albeit it was suggested that this pathway could also be used in an electronic format) for the care of pwMS at a hospital in Spain (29), and a logic model (implementation yet to be explored) for the design of clinical pathways for the identification and management of cognitive problems in pwMS (30). Considering that patient pathways are a better fit for MS due to their more holistic perspective and with digitalization on the rise, the concept of pathways may now well become more popular within the domain of MS. One key feature which might prove beneficial there is variance analysis.

Zander described its importance as follows (21, 31): “Real progress in healthcare practice will not be made until variances from measurable intermediate goals and patient responses and outcomes are combined with other data and transformed into knowledge.” This is to say that a pathway also serves as a benchmarking tool. It allows the care team to detect any deviations for a given individual patient from procedures and outcomes that have been defined as standards or milestones along the pathway for a specific homogenous patient population. Retrospective pathway data then can also be used for the further investigation of the potential reasons for observed variances. Among others, Du, Huang and Zhou (32) conducted a review of 496 papers on pathway variance research, covering a 25-year time period from 1994 to 2018, and provide a comprehensive overview of different approaches that can be used for variance analysis.

As Zander (21) points out, assuming a bell curve, a 32% variance is always to be expected as well-designed pathways then ought to reflect patterns in care provided to approximately 68% of discreet patient populations in relation to a given disease and its treatment across time. Variance analysis is a key function of pathways as it is crucial for quality management and thereby for the standardization and harmonization of clinical practice and healthcare services across all disciplines and providers involved (20).

A list of all pathway characteristics touched upon in this section and examples derived from a comprehensive literature study can also be found in De Bleser, Depreitere et al. (16). From our perspective, however, some of the characteristics mentioned by them can rather be classified as functions than as characteristics (e.g., management of patient care and variance analysis).

### 2.1 Next level: pathways going digital

In the past, pathways used to consist of multiple paper-forms, including preprinted order sets, a documentation system for outcomes, a variance reporting system, and frequently, patient education materials. For the organization of entries two designs were common: the *matrix design* similar to a Gantt chart and the *algorithm design* similar to a decision tree (33). The matrix design essentially corresponds to the *chain models* described by Vanhaecht, Panella et al. (15) who further stated that pathways could also come in the shape of *hub models* or *web models*.

Nowadays, data previously recorded on paper are either digitized *post hoc* (ideally automatically using optical character recognition applications) or are recorded via digital applications on electronic devices *a priori*. This also affects pathways in healthcare as thereby digitalization sets in and changes *how* data can be generated, collected and used. It brings about fundamental changes in *how* information on a patient’s health state can be gained, saved, retrieved as well as merged and analyzed in conjunction with relevant context information, e.g., on a patient’s lifestyle or that of other patients with the same disease and similar characteristics.

Digital information from pwMS can be stored in virtual databases. Virtual databases may allow the automatic integration of complex multimodal information with AI. This might include not only brain imaging and regular neurological examination, but also data from wearables, ambient sensors or omics (e.g., metabolomics) (34). Some information, such as the date of recording, can even be added automatically by the computer (e.g., the time and date when a patient’s information is being changed or updated).

Moreover, in consequence of digitalization, some steps along the pathway that could only be carried out at a certain time (e.g., within consultation hours) and place (e.g., hospital) in the past may now be completed virtually and even from remote (e.g., collecting patient reported outcomes) while other steps still require face-to-face contact (e.g., taking of blood samples) and can only be digitalized in part (e.g., electronic submission of blood work results). This, in turn, suggests that healthcare processes now overall may comprise even more activities due to digitalization: Telehealth interventions such as virtual meetings with a physical therapist and virtual consultations with a neurologist may now complement regular inpatient and outpatient appointments. Hence, the range of health care services may be extended.

On the other hand, any such service (e.g., regular check-up) usually relies on the execution of more than one process which again consist of multiple steps (scheduling of appointment, attendance of appointment, any tasks that need to be carried out to complete the check-up itself, follow-up tasks such as handing out of prescriptions and letters of referral etc.). Therefore, at the same time, digitalization may also lead to a reduction in the number of steps needed for the completion of any processes that might belong to a service (e.g., online transfer of prescriptions rather than printing out prescriptions, giving them to the patient who then may take them to the pharmacy where they are scanned before the patient is given the prescribed medications). Hence, the number of steps needed for a particular service may be reduced.

Researchers describe this integration of telehealth interventions into standard care as “hybrid telehealth model” (35–37) or, in general, speak of “hybrid models” (38, 39) or “hybridization” (40). Phrasing it this way highlights the fact that physical face-to-face interaction neither is nor will be entirely be replaced by digital means. Conducting a literature review of telehealth interventions studies in the field of MS, Xiang and Bernard (41) stated that telehealth interventions had been found to reduce missed work days as well as travel costs associated with follow-up appointments and that patient and healthcare providers overall were satisfied with their utilization while disparities in the access to virtual tools amongst vulnerable populations and completion of neurological exams from remote were still posing a challenge. Telehealth interventions studied and used so far include, e.g., MSCopilot (42) and MS Sherpa (43). A comprehensive list and discussion of potential telehealth interventions applied in pwMS can be found in (1).

Despite all progress made in this regard, care will partially still have to be provided on site and in person outside of the virtual space and through humans, not machines. But in light of ongoing digitalization efforts, the functions of the traditional paper-based pathway concept in healthcare can be upgraded.

Organizations have only just started to adjust their pathways accordingly in recent years, and researchers have increasingly presented new approaches for pathway development considering the changes brought about by digitalization and new technological advances such as AI. Again, different terms are being used in this context: E.g., “digital health pathway” (44) or “digital care pathway” (45–48), “partially digital pathway” (49), “human-centered integrated care pathways” or simply “integrated care pathways” (50), or “digitally enabled care pathway” (51–53).

**Table 2** provides a comprehensive, albeit not exhaustive overview of corresponding changes in selected pathway functions (traditional paper-based vs. digital pathway) along with potential benefits and drawbacks.

**Table 2:** Traditional paper-based pathways vs. digital pathways.

(16)	Traditional paper-based pathway	Digital pathway
Access to pathway contents	• Paper-based: This may complicate information exchange between stakeholders, which in turn may limit stakeholder integration (56). • Pathways tend to only refer to a specific episode of care (18), only involving stakeholders of one and the same organization, which may also limit stakeholder involvement. • The number of stakeholders which can use the pathway or be involved in pathway definition is limited by the use of paper. • Physical access required (56): Pathway information needs to be copied or physically transferred for information exchange. At least in the first place, i.e., at the place of recording.	• Digital format: Information can digitally be transferred and exchanged between stakeholders. • Supports digital processing of data which enables the automation of some tasks which previously required human labor. • Allows for the digital and even semi-automatic or fully automatic integration of information. • Democratization (57): The group of pathway stakeholders can be extended more easily, e.g., active involvement of patients since electronic devices such as smartphones are mass-market consumer goods by now and internet access is widely available (58).
Evidenced-based care	• Scientific insights need to be manually integrated into the pathway by responsible team members. • This requires self-initiative from team members/their organizations to check appropriate source for updates of professional guidelines, new scientific insights on a regular basis.	• Potential sources of new scientific insights (e.g., public scientific databases, official regulations and professional guidelines that are being published electronically) can be linked to the pathway. • Responsible team members can be automatically alerted to relevant information retrieved from these sources. • Even the automatic integration of relevant information (as far as sensible and reliably doable with available technology), i.e., automatic update of pathways becomes possible.
Chronology of events	• Pathways are fixed, i.e., pathways do not automatically adjust when a patient differs from the pathway that is in use. Potential changes that may become necessary due to such deviations are not foreseen by definition, i.e., not defined on paper. • Prone to human recording mistakes (e.g., forgetting to indicate the time/date of a patient visit due to stress). • Multiple paper forms (e.g., orders for medication sets and tests, laboratory reports etc.) containing information belonging to a patient’s pathway (33) or even single pieces of information from those must be matched and sorted by hand if a chronology of all events is required. • Risk of incomplete information due to missing relevant information: blank in chronology of events.	• Time and date and other additional meta-information considered to be essential (e.g., name of the person in charge) can be added automatically. • Chronological order of events may be determined automatically. • Integration of pathway information from different sources provides a more complete picture of events. • Through the implementation of interoperability standards and corresponding interfaces, pathway information from multiple different sources (e.g., recorded by different stakeholders) can even be integrated automatically. • Ideally, a more complete picture of events can be obtained this way: complete chronology of events.
Inventory of actions	• Once a pathway has been defined, there is no flexibility to quickly add additional interventions not foreseen at the time of pathway definition by the responsible team. • In general, the inventory of actions that can be used for the definition of a pathway is limited due to the limited amount of space on paper.	• Through the use of AI, an updated version of the prospective pathway for a specific patient could be provided automatically based on advanced analysis of variance and patient population data. • The size of the inventory of actions is basically unlimited as relevant activities could be displayed or discarded on screen as needed, plus scrolling and browsing is an additional option on screen to fit in more activities.
Management of patient care	• Information exchange and thereby the coordination of activities heavily relies on the exchange of papers or paper copies and scans (paper-based patient files). This is highly labor-intensive and time-consuming. • Keeping track of information and gathering potentially relevant information from outside stakeholders is complicated. Pathways therefore tend to only refer to intra-organizational settings or one episode of care (20, 25), i.e., the coverage of the patient’s journey is limited. • Risk of redundancy: Although this risk is already reduced by the use of pathways in general, there is still a risk that activities carried out multiple times for a lack of knowledge or due to lacking standardization and interoperability.	• Exchange of information is facilitated by means of electronic communication. • Albeit the use of interoperability standards and corresponding interfaces is highly recommended (59). A lack of such poses a barrier. Implementing such standards/interfaces also requires the investment of time and financial resources. The return of investment on the other hand (in terms of money but also in terms of informational gains) highly depends on the initiative of the stakeholders involved. The more stakeholders decide to make the effort, the higher the return on investment for all actors involved. • Presuming interoperability, the risk of redundancy can be reduced through the electronic recording, storing and exchange of information.
Variance analysis	• The detection of deviations from the pathway and finding possible explanations is mostly left to the HCPs using the pathway (20). This makes it prone to human errors. • Relevant information can easily be missed or also go missing when everything/much information is recorded on paper. This can lead to mistakes in the analysis of variances. • The quality of analysis and interpretation of results very much depend on the expertise and experience of the person in charge.	• Semi-automatic or automatic recording and detection of deviations becomes possible. • AI can support HCPs in identifying possible reasons for deviations. • Any program is only as good as the algorithm behind it: Errors in programming can lead to false analytical results. • The same goes for the use of machine learning or AI: Computers do not “think” like humans, they only calculate. • Human expert knowledge can be translated into algorithms. This may benefit, e.g., less experienced HCPs. This especially poses an advantage when there is a shortage of experts (e.g., fewer specialized physicians in rural areas).
Education of patients and care providers	• Prior to digitilization, pathways were mainly drafted for and used by clinic staff. • Limited reach: Reaching of potentially relevant stakeholders has also been complicated as analogue education materials (e.g., handouts, CDs, DVDs) cannot as easily be spread. • Interactive education requires physical presence (e.g., doctor-patient workshops on site).	• Comparably more stakeholders can be reached as physical access is not required anymore. • Currently, especially the number of older people who do not know or do not want to use electronic devices (e.g., smartphones or tablets) might still be relatively high. On the other hand, older people are more likely to be sick and therefore would also be among those who would primarily benefit from the use of pathways in healthcare. In this regard, the digitilization of pathways could also pose a barrier in communication that would have to be overcome. However, chances are that this issue will resolve over time as younger generations who have been raised in a digital world grow older. • Education becomes independent of time and location (25). • Interactive elements (25) can also be included in digital formats (e.g., using quizzes, virtual reality, touch screen functions) which might further increase patients’ adherence.
Communication	• Improves communication between stakeholders as it supports the exchange of information, e.g., use of standardized paper forms. • Reduces loss of information by guiding HCPs in their routines. • Translates professional guidelines, scientific recommendation, and official regulations into practice by adopting them to local structures. • Promotes team work by easing communication through the support of information exchange, e.g., change of hospital staff between shifts (20).	• Electronic devices offer more opportunities for communication (58). This facilitates the sharing of pathway information and opens additional channels of communication, e.g., direct information exchange between caregivers becomes possible, no need for the patient to carry papers back and forth. • Can facilitate patient-caregiver/doctor communication. Everyone having access to the pathway is kept in the information loop. Hence, everyone is up to date what has happened and knows what needs to happen next.
Data collection and storage	• Purely or mostly paper-based: time- and space-consuming, labor-intensive. • Lacking standardization and harmonization due to human errors complicates data exchange and reduces comparability. • Lack of data interoperability: information can only be matched and merged by hand. • Interpretation of data strongly depends on the expertise and experience of the person in charge. • Analogue media such as printed images or radiological scans, text may be compromised through storage (e.g., damaged by sunlight or water or in the process of transport or copying). • Risk of data loss in the process of information exchange/transfer between stakeholders.	• Provides a structure for digital data collection and thereby supports the standardization of documentation. • It may allow for the automatic check of entries which also benefits the standardization of documentation and thereby promotes interoperability and supports information exchange. • Computers/servers consume less space than paper files. • Can be more time-efficient dependent on the usability and design of software applications in use. • More energy-intensive in terms of electrical power needed for the use of electronic devices. In particular, the training and use of AI requires a lot of computing capacity and therefore a lot of energy. • Easier to backup. Backups can be created automatically. Therefore, the loss of data can be avoided more easily. • Risk of hacker attacks. • Facilitates the integration of multimodal data. Thereby more data becomes available for the purpose of variance analysis.

Source: Self-prepared, loosely based on (54, 55).

In conclusion, digital pathways can be used to *virtually* track when, where, and what types of data containing relevant patient information are being created by whom (using which instruments and applications) in the care process as part of the patient’s journey through the health system. In addition, digital pathways can also provide *virtual* guidance to patients and HCPs alike about what ought to be done next. Providing a defined sequence of activities and events as guiding reference is also a key function of their traditional paper-based counterparts, but by means of software design and programming, *navigating* pathways in the digital sphere becomes comparably more fluid: E.g., automated searching for a certain piece of information in a virtual database with a computer is much quicker than going through individual paper records by hand, and events can be automatically ordered by time and matched and compared to those of a given standard pathway (pathway template).

### 2.2 How to: the digitalization of pathways

Up until now, however, the number of examples for digital pathways that can be found in the literature is still fairly low. Examples we found refer to conditions such as arrhythmia (45), acute kidney injury (51–53), HIV (60), genetic testing for breast cancer (49) and Parkinson’s Disease (53). But, as far as we know, a digital pathway for MS, or an approach for how such a pathway could be built has not been put forward yet.

In general, *connected health* “where patient-centered care results from following defined healthcare pathways undertaken by healthcare professionals, patients and/or carers who are supported by the use of health information technology (software and/or hardware), regulated when used as a Medical Device, and facilitating appropriate health data sharing” (59), is still far from being what many of us experience in our daily lives. A very recent report of the Organization for Economic Development confirms that: “[ … ] innovative ways of delivering healthcare and supportive ICT [information and communication technology] are typically deployed as pilots or research projects, with project specific funding” and “[ … ] many digital tools are not scaled to reach a larger population even if they are successful or show promise” (61).

Recent advances in AI technology, however, might change that as these advances have also steered policy makers around the globe to direct their attention to the potential and risks of this technology for all kinds of industries including healthcare, see, e.g., the recent negotiation of the AI Act within the European Union and a recently study prepared to assist Members and staff of the European Parliament in their parliamentary work (62).

In general, AI as such refers to an algorithm that mimics the cognitive skills and behavior of humans. Machine learning (ML) is a subset thereof and comprises data-driven algorithms that enable machines to learn from data while deep learning, on the other hand, is considered a more advanced subset of ML as the data-driven models used for it resemble multi-layer neural networks (63, 64).

**Figure 2:**
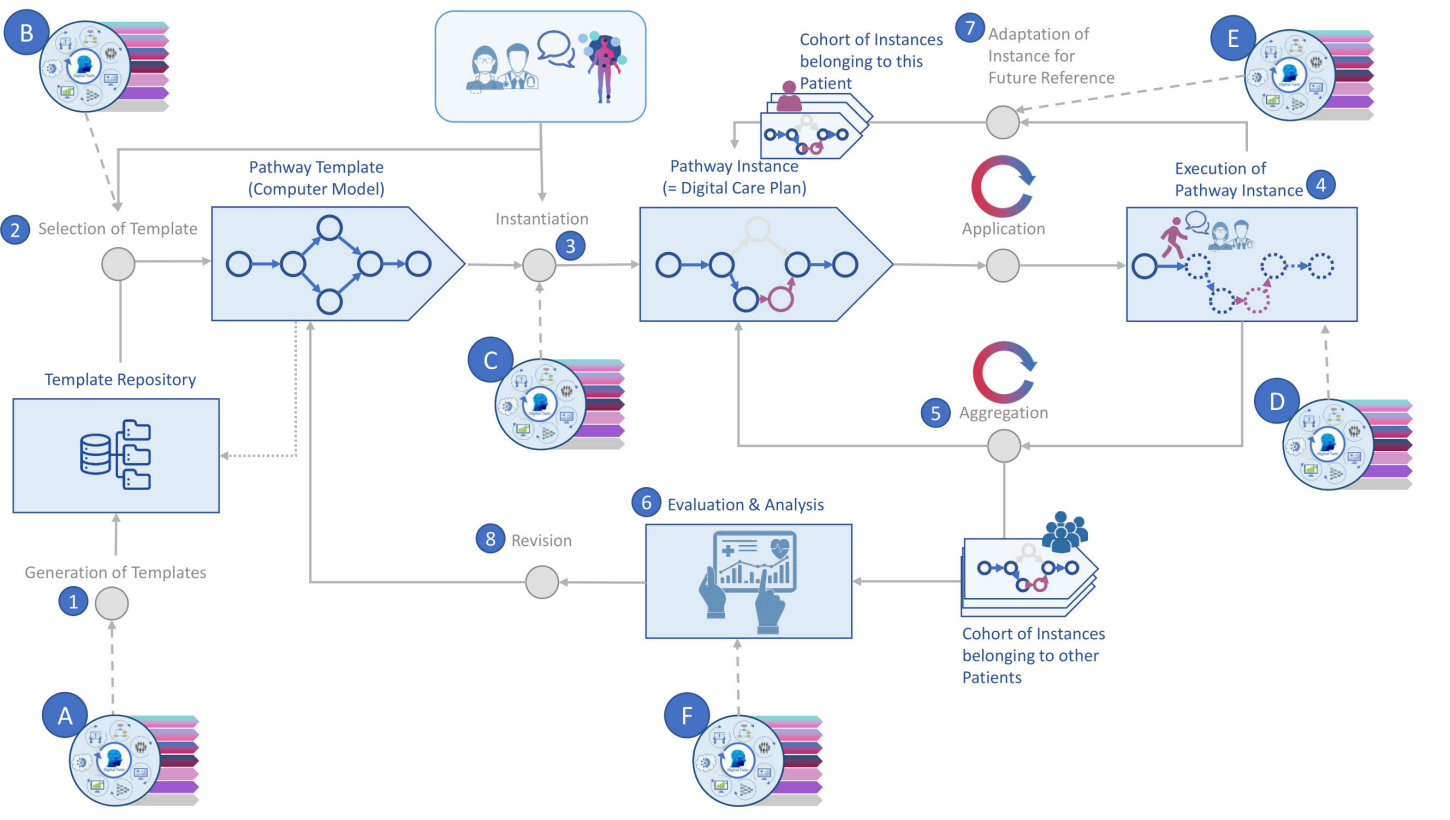
Pathway modelling supported by AI. Source: Self-prepared, using icons created by Freepik – Flaticon, https://www.flaticon.com/free-icons/ and Vecteezy, https://de.vecteezy.com/gratis-vektor. For a detailed description of this figure, please refer to **Table 3**.

AI technology cannot only be applied in telehealth interventions, but also in the modelling of digital pathways (see **Figure 2** along with **Table 3**). E.g., Schlieter, Benedict et al. (65) propose a conceptual framework for the design and setup of personalized dynamic pathways in the digital sphere. Their reference architecture for the integration of such pathways into given health information systems enables the ongoing dynamic adaptation of pathways to the specific personal needs of patients and caregivers for decision support, education and recommendations.

**Table 3:** Description of pathway modelling and involvement of AI therein.

Activity	What happens and how can AI support this activity?
**1**	Pathway templates are generated from collected retrospective data and standardized process knowledge (e.g., professional guidelines). **A:** AI technology can generate suggestions for templates by clustering available patients into patient groups based on retrospective information on the sequence of procedures and respective outcomes.
**2**	The treating HCP and their patient jointly select a fitting template and… **B:** AI can help by identifying a patient’s subtype and based on that offer the best-fitting templates for selection.
**3**	… make adjustments as needed. In doing so, they create a patient-specific pathway, i.e., a digital care plan (pathway instantiation). **C:** AI can highlight what steps indicated in the selected template will likely have to be changed based on the individual profile of the patient at hand (e.g., because of existing co-morbidities, medication intake etc.) AI can also help to optimize the selected template regarding the sequence of procedures that will have to be performed taking into account given available resources (e.g., staff and rooms).
**4**	The execution of pathway instances in real life (provision of care, e.g., by the MS care unit, according to the patient-specific pathway that was created). **D:** AI can be used, e.g., to automatically deploy relevant questionnaires for the collection of PROMs to the patient via a patient app or to send an alert to HCPs and patients in case of unwanted outcomes.
**5**	Data produced by pathway execution is aggregated for…
**6**	… the analysis and evaluation in conjunction with existing pathway instances from other patients using the same template. **F:** AI can make suggestions for the revision of templates taking into account the newly aggregated data.
**7**	In parallel, the instance that was created in the first place is updated to mirror what was done and happened in the real world during pathway execution compared to what was expected to be done and happen. As a result, an adapted instance is created for future reference and added to the cohort of instances belonging to the patient at hand. **E:** AI can help to predict what should happen next, in order to obtain the best-possible outcomes for the patient at hand in the future.
**8**	Following from the evaluation and analysis in **6**, the pathway template that had been used may be revised.

Source: Self-prepared.

Pathway modelling coupled with AI technology can be broken down into eight core activities (65, 66). Thereof 1) only takes place occasionally (e.g., substantial changes in professional guidelines) while 2) and 3) only refer to newly diagnosed patients or patients visiting the facility where digital patient pathways are being used and modelled this way for the first time. **Table 3** provides a short description of the relevant activities.

The application of this modelling approach yields pathways that support the intra- and interorganizational management, standardization and harmonization of all patient data that are being generated along the pathway across multiple data sources by multiple stakeholders. A specific digital patient pathway then provides insights in how different procedures are carried out in a specific healthcare setting and enables the *virtual* integration of multimodal data. In consequence, it also allows for the structured assessment of every step that any one patient takes on their (path)way through the healthcare system, i.e., the evaluation of any health care services that have been or are planned to be carried out. This, in turn, is essential for quality management.

## 3 Wanted: digital pathways for MS care

The digitalization of MS care, including the development of digital pathways, may lead us to better grasp and handle the disease’s complexity and could help us to overcome related challenges in MS care. The interplay between neuroinflammation and neurodegeneration which varies for each individual patient leads to heterogeneity in symptoms, course and outcomes in pwMS (67). This is what makes MS diagnosis and treatment particularly challenging.

Even though the introduction (in 2001) and recurrent revision of the McDonald criteria (in 2005, 2010 and 2017, respectively) have significantly shortened the time to diagnosis and improved sensitivity overall (68–71), those criteria need to be applied with caution as they were not designed for the differential diagnosis of patients, but rather to identify MS or the likelihood of manifesting MS in patients presenting with a clinical isolated syndrome (CIS) (72, 73). Despite all progress made in this regard, an extended time to diagnosis from the onset of symptoms is still common (74–76), and MS misdiagnosis also still poses a problem in practice (6, 77–79).

According to the authors of the Brain Health Report, diagnostic delay is primarily caused by inadequate access to specialist healthcare (i.e., a low per capita number of neurologists and lack of diagnostic tools such as MRI scanners) and insufficient awareness of the disease among family members and primary care physicians. Atypical symptoms (76) or older age at the onset of disease (75) may additionally contribute to diagnostic delay. Ultimately, delays may occur at two points in time along the pathway: One the one hand time may be lost until a patient finally decides to seek professional help. On the other hand, it then may take even more time until a specialist finally diagnoses MS in a patient (80).

To counter these challenges, researchers and MS professionals advocate the establishment and implementation of quality standards and recommend that specialists with expertise in relevant functional domains should be involved in MS care (81, 82). Digital patient pathways can help put these recommendations into practice as they can support the management and coordination of MS care across the whole care chain. For this purpose, e.g., teams from specialized clinics (also called MS care units [82)] may be put in charge of the overall administration of digital patient pathways. Members from these teams can virtually share their knowledge and experience, e.g., first of all, by defining a set of standard pathways, and, second, by providing their colleagues and patients following a pathway with virtual counselling services (e.g., via virtual notes, video sessions). In doing so, the team may oversee MS management and treatment from afar and on site.

There is a large body of MS research available as well as several evidenced-based medical national and international guidelines [e.g., (83–85)] which would jointly constitute another valuable source of content for pathway modelling (see also *Section 2.2*). Resulting digital patient pathways could provide their users with the specialist knowledge gained from all of these sources and could thus also be used to educate and train general neurologists and MS experts in the correct application of diagnostic criteria and MRI interpretation to avoid misdiagnosis as suggested by researchers (78).

A set of predefined standard pathways in the pathway repository, enriched with scientific findings from accredited sources forms a pool of machine-readable knowledge. Everyone who has access to a digital patient pathway thereby also gains access to this knowledge, and to the specialized team administering it. What makes using digital patient pathways worthwhile though is their flexibility. Looking at the next steps suggested, a neurologist may still decide to change the pathway and, e.g., initiate a different type of treatment than was recommended by the pathway. As depicted in **Figure 2**, with the help of AI this change of plan can be compared against what happened in other cases, such that the prediction of the further pathway will be adopted accordingly.

Even without the added benefits of AI in terms of predictions and without the AI-supported automatic adjustment of suggestions, predefined digital standard pathways by themselves provide a virtual structure, which may guide HCPs and patients in their doings, offering significant advantages over paper-based pathways (see **Table 3**). Their virtual structure may help HCPs and patients (1) better grasp the disease and treatment strategies in their complexity by keeping them focused in the present while enabling them to take a step back and take a look into the past and future when needed, simply following the pathway in either direction. As digital patient pathways, if used properly, also govern data collection, all one needs to know is virtually available in one place: expert knowledge as described above, aggregated health data from MS patient populations and health data of the individual patient of interest. Connecting these different pools of knowledge and running further analyses over the data available has not been possible in times of paper. But through this virtual connection and thereby through the integration of competences, digital patient pathways may eventually (2) speed up diagnoses, (3) support the timely initiation of interventions and (4) patient involvement in the decision making process, (5) facilitate monitoring, (6) counter the shortage of MS neurologists, (7) help minimize risks and may (8) even contribute to the post-marketing refinement of drug profiles.

One major benefit of digital patient pathways is that they can promote the standardization and harmonization of MS care by providing a virtual structure for everything that needs to be done. By applying the modular approach from Section 3.1., digital patient pathways may still be manually adopted in an agile manner. This requires a virtual dashboard application, but not necessarily the use of AI.

Integration of competences means that digital patient pathways also constitute a virtual link between patients and their caregivers – everyone having access to the virtual dashboard application where the pathway is displayed can tap on its embodied knowledge, but also contact service providers that have been linked to its modules, i.e., the individual steps belonging to that pathway. Hence, overall access to specialized care can be improved through the inclusion of online services from specialized clinics (e.g., second opinions on MRI scans, test results etc.; online consultations) in the pathway. Digital patient pathways thereby would provide a platform and structure for MS-related communication and education which might also help increase general awareness of the disease.

Even though diagnostic delay is a common problem, pwMS are on average still comparably young at diagnosis, i.e., 20 to 40 years old (67, 86), and their median life expectancy is estimated to be only about 7 years lower than that of the general population (87, 88). Hence, most patients who have recently been diagnosed with MS will usually go on to live with the disease and its symptoms for many decades. This leads to a huge amount of *multimodal data* [see, e.g., (89)] being accumulated over time for each patient, including, among others, MRI scans, laboratory test results, results from cognitive tests, values recorded via gait and jump analysis.

All of these data characterize the individual state of disease in a given patient. Collecting relevant information from different sources (e.g., other HCPs), sorting and ordering all this information for one single patient can already pose a challenge and contribute to delays in diagnosis and treatment in practice. Moreover, the number of features (variables) recorded for one individual patient may also well exceed the total number of patients in a given MS dataset, or, in more general terms, the number of features available for one data record may well exceed the total number of records included in a dataset.

Such *high-dimensional datasets* are often sparse, noisy, cross-sectional and lack statistical power. This is even more likely to be true for high-dimensional datasets comprising multiple modalities. Traditional data analysis methods are not fit to cope with these kinds of datasets. ML and AI-based approaches supporting big data analytics on the other hand can help tackle these problems (90). But for the application of such, data need to be available in a machine-readable format and need to be collected in a standardized and structured manner – one of the key functions digital pathways have to offer.

Despite posing analytical challenges, the multimodality of MS data can also be considered an advantage. Regarding the interpretation of image data in general, it has been shown that results improve when practitioners have access to clinical and laboratory data which provide them with critical context information (91, 92). The availability of additional information is not only deemed important by radiologists (93), but also affects image-data interpretation in other disciplines such as pathology, ophthalmology, and dermatology (94). Huang, Pareek et al. (95) conducted a systematic literature review and found that the same was true for the application of AI/ML in the medical field as multimodality fusion models exhibited an increased accuracy of 1.2% to 27.7% compared to single modality models when used for the same task.

The number of data records in the dataset(s), i.e., data volume, and modalities included, determines what ML technique and what algorithm can be applied. With regard to data volume the following typically holds true: The higher the sample-to-feature ratio (the number of data samples divided by the number of features), the easier it will be to obtain any meaningful results through the use of ML, in particular supervised learning. Hence, combining multiple data sources and techniques as well as tracking and analyzing of ML metadata which are being generated in the process to validate the usefulness of generated models is highly recommended (90).

All of the challenges presented in this section call for new approaches to improve MS diagnosis and treatment. And digital pathways could literally lead the way to the application of big data analytics in MS care which eventually could help us to overcome these challenges. In the following sections, we thus present our ideas for a modular-integrative framework for the building of digital pathways in MS.

### 3.1 Piece by piece: a modular service portfolio for digital pathways in MS care

The idea of modularity was originally confined to the manufacturing of goods, but the idea has long spread into services. Following the concept of modularity, a product or service can be split up into individual standardized components, i.e., modules, which then may be mixed and matched together to obtain a functioning final version of the product or service in question. Each resulting module needs to constitute a self-contained unit such that it can be shuffled around or skipped as desired when building the final version (96). Modularity thus facilitates process management. On top, it allows for the customization and personalization of products or services but does not require one to start from scratch (97–99).

Meanwhile, the concept of modularity has also been applied to healthcare services. Recently, Peters and Richter (100) conducted a literature review and survey for the modularization of healthcare services. Taking into account given process dependencies, Peters and Richter (100) finally presented a list of thirteen modularization parameters, grouped into four categories. For each parameter, they provided an exemplary question through which respective process dependencies could be identified, e.g., for patient needs dependency: *Do the processes contribute to satisfying the same patient need?*

**Figure 3:**
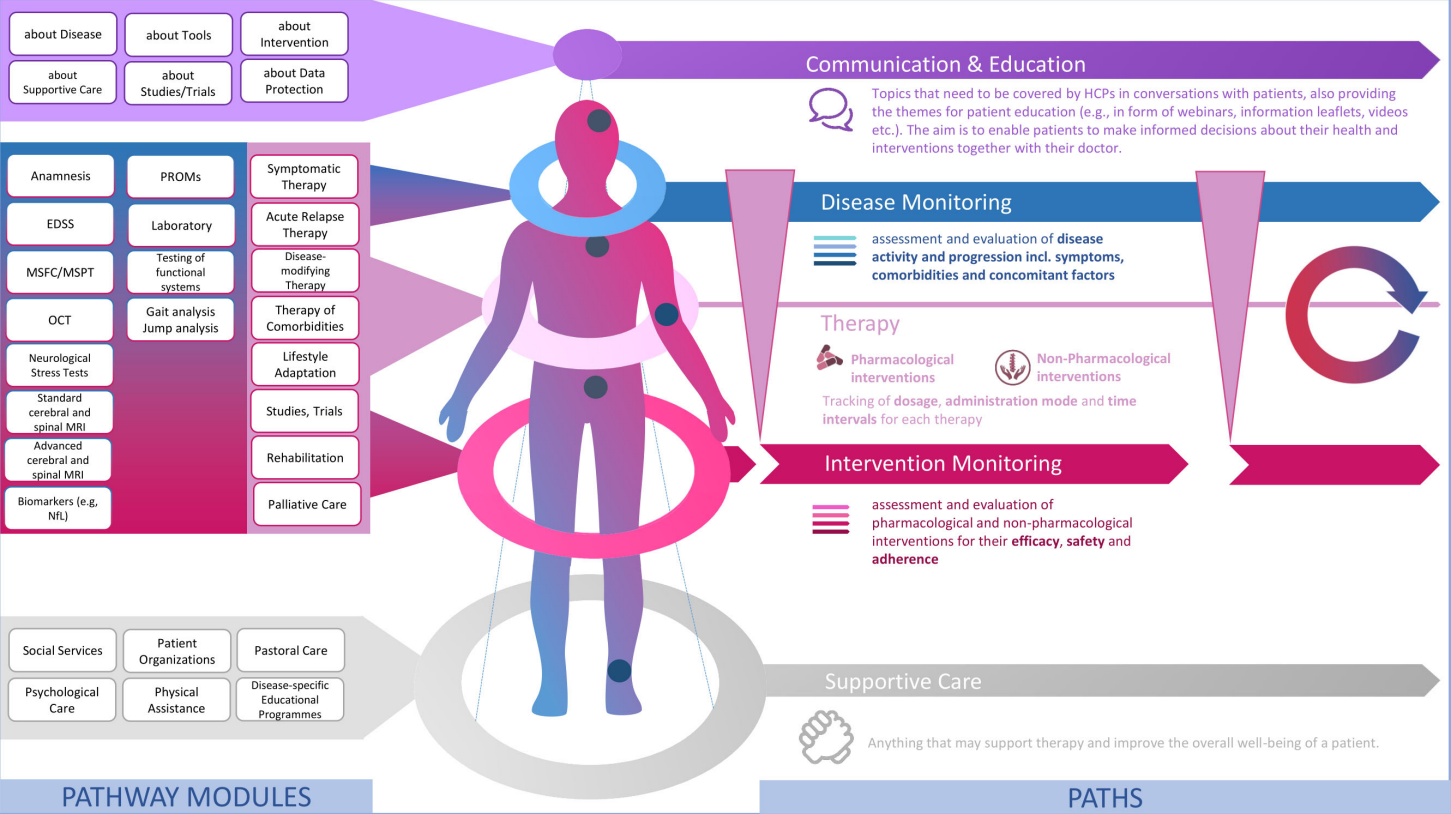
A modular service portfolio for the design of digital patient pathways in MS care. Source: Self-prepared, using icons created by Vecteezy, https://de.vecteezy.com/gratis-vektor.

Mirroring the approach presented by Peters and Richter (100), we have identified different health care services which are relevant for MS patients. Following the example of Peters and Richter (100), those services can be grouped together forming a modular service portfolio (**Figure 3**). We propose that those categories themselves can be seen as parallel paths, implying that a patient’s pathway can be separated into different paths as services belonging to the same category could also be tracked along one and the same path. Basically, this constitutes a filtered view on the patient’s pathway, setting the focus on the chronological sequence of services for a specific category and the longitudinal development of corresponding data input values (e.g., choice of medication, dosage etc.) and values of outcome measures (e.g., EDSS scores, MRI data).

### 3.2 Paving the way: the building blocks of digital pathways for MS

Modules of the same category serve one common, dominant purpose as outlined in **Figure 3**. Equally, modules of the same category are characterized by similar process dependencies, e.g., in terms of location and stakeholders. Modules in the top category *Communication & Education* include any services which aim to inform and educate patients on MS and all aspects relating to their life with MS and anything they may encounter in the course of MS treatment. Therefore, modules of this category address patients’ needs for information and education.

Then there is a number of modules which are made up of the diagnostic tools that may be used for the monitoring of the disease itself and that of any interventions. Hence, there are two distinct monitoring paths along which related modules can be tracked: *Disease Monitoring* and *Intervention Monitoring*. From our perspective, this distinction allows for a more systematic and agile monitoring, paving the way to precision medicine in MS care. Modules of this category equal the structured set of components from our monitoring matrix (101) and are derived from evidence-based guidelines for MS treatment which mostly takes place at a specialized MS clinic, hospital or at a neurologist’s office. I.e., those modules mainly share healthcare system-related and general dependencies.

Intervention Monitoring, in turn, is tightly linked to therapy. The category of the same name comprises different types of *Therapy*. Along this path, dosages, administration mode (self-administered vs. administration by medical staff; oral, infusion, injection etc.) and time intervals can be tracked. Again, modules of this category are highly healthcare system dependent as the prescription of any kind medical treatment is strongly governed by national regulations and public authorities such as the U.S. Food and Drug Administration (FDA) and the European Medicines Agency (EMA) overseeing the approval of medicines for marketing.

At the bottom then follow services which do not necessarily require medical expertise but may still have a positive effect on the overall well-being of patients, i.e., may make a positive difference in a patient’s life with the disease and support MS therapy. As the impact and potential benefits of such *Supportive Care* should not be neglected in MS care we argue that these should also be integrated in digital patient pathways. Modules of this category again strongly cater to patient needs and heavily depend on patient engagement and personal encounters with service providers.

When it comes to process modeling, services represent the procedural operations of health care providers and are also referred to as activities. With regard to the MS use case at hand, the service modules included in the modular service portfolio in **Figure 3** represent a set of activities or reusable (sub-)processes. As outlined in Peters and Richter (100) with reference to (102), in general, each module thereby contains at least one activity which is carried out to provide patients with the service it stands for.

If a patient visits a healthcare facility, a number of activities are usually performed by different actors (e.g., administrative and medical staff). The visit itself is the *(start) event* which triggers these activities. The sequence of activities may be determined by interdependencies with other activities (103), e.g., IT and general infrastructure of the facility, availability of staff and rooms at the facility. Among others, AI technology can help find the optimal sequence of activities taking into account given constraints like these (see Section 2.2).

There is a wide range of activities which may take place in a healthcare setting. With regard to activities specific to MS care see, e.g., the modules which refer to typical clinical and paraclinical procedures used for MS monitoring such as the application of the Expanded Disability Status Scale (EDSS) or magnetic resonance imaging (MRI). In the literature, a broad distinction is drawn between clinical processes (e.g., disease and intervention monitoring) and organizational processes (e.g., administrative processes such as billing). Even though our exemplary MS service portfolio does not explicitly refer to administrative processes (activities) such as the registration of patients at the counter and the writing of prescriptions, those naturally occur and may be carried out as part of the modules listed in **Figure 3**. Besides that, one could also add another category including modules referring to the general administration of MS care. Modules of this category could include activities (e.g. billing, bookkeeping etc.) which need to be carried out for each of the modules listed in the other categories and could thus be aggregated to form a module of their own (e.g., accounting).

### 3.3 Filling in the blanks: adding another layer of information

Well-designed health information systems can be configured to automatically add and store meta-information in database tables (*event logs*) for later analyses. The so-called process execution data may contain some basic information such as what particular activity was performed when and by whom (e.g., which staff member), concerning which patient. Hence, event logs show what happened when the process was executed in reality. Insights gained from the analysis of event logs, i.e., from *process mining*, can be used for the creation of new and the optimization of existing processes (104, 105) and therefore provide valuable insights for quality management and the refinement of pathways by means of process engineering (106).

This is why we propose to also specify corresponding attributes according to local conditions for any modules that are to be included in the final modular service portfolio. From our perspective this would increase the value of the portfolio as it would likely support the subsequent translation of modular pathways into a modeling language such as BPMN for the development of a virtual dashboard for instance.

Modeling languages have commonly been used for the graphical depiction of pathways, defining the logical sequence of events and activities of a process, e.g., for software programming. The use and interpretation of such languages, albeit to a variable extent, requires expert knowledge whereas modular building blocks can easily be pieced together to quickly draw up a specific patient pathway. Peters and Richter (100) therefore conclude that resulting modular representations of patient pathways might also be easier to grasp for patients. Along this line, we propose that our modular-integrative framework could also enhance doctor-patient communication and thus support shared-decision making in MS care.

**Figure 4:**
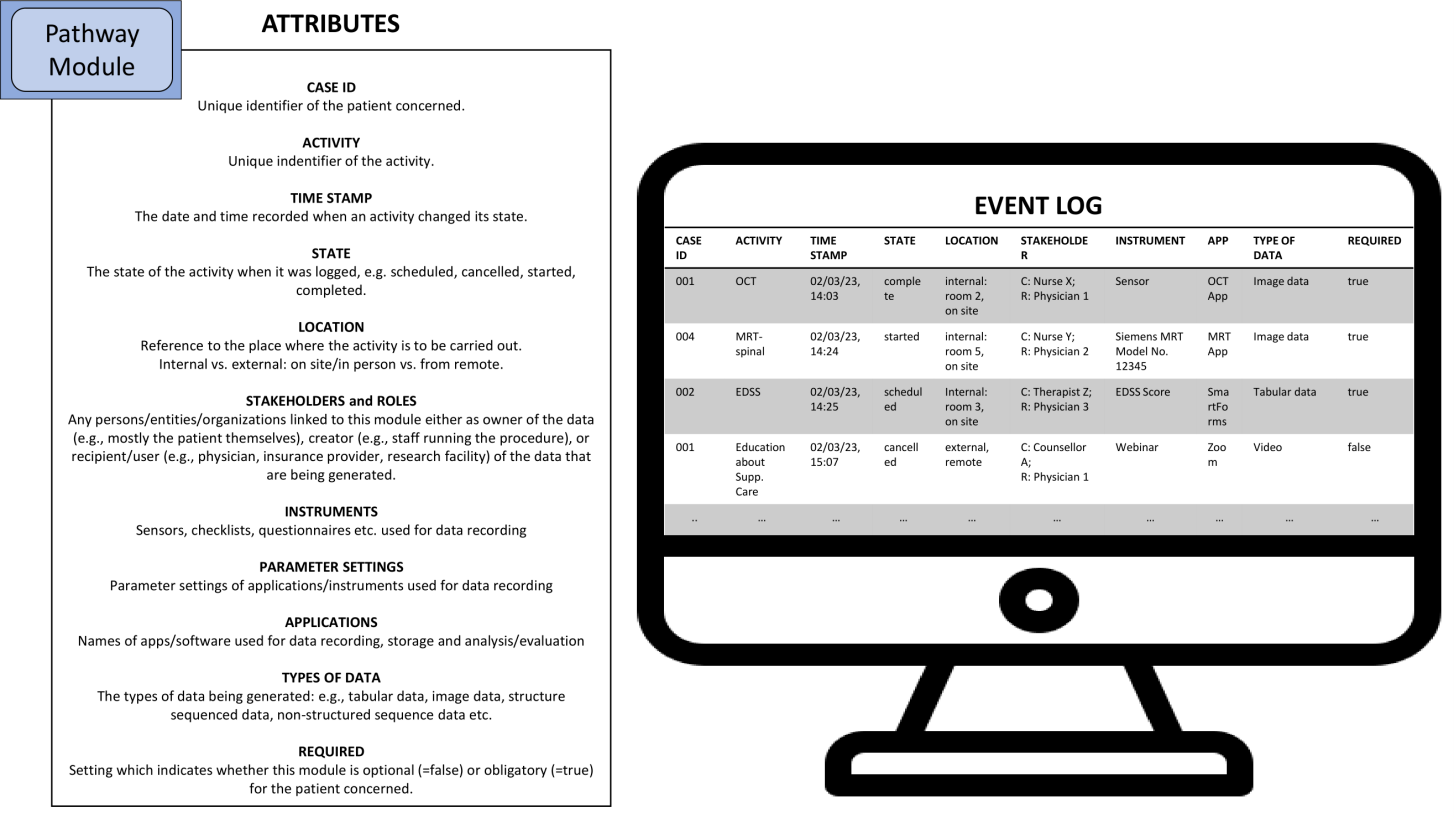
Possible attributes of MS pathway modules which can be used for the generation of event logs (C=Creator, R=Recipient). Source: Self-prepared, using icons created by Freepik – Flaticon, https://www.flaticon.com/free-icons/.

In **Figure 4**, with reference to De Roock and Martin (104) and Munoz-Gama, Martin et al. (105), we further list possible attributes which could enhance the utility of pathways. Moreover, we find that the modularization parameters suggested by Peters and Richter (100) could also be used to identify suitable attributes which should be recorded by information systems supporting healthcare as corresponding attributes largely overlap with the contents of the modularization parameters they describe in their paper (see Section 3.1).

Hence, if a new health information system is to be chosen or is even specifically being developed, considering which attributes should be stored in event logs in the first place can greatly enhance the variance analysis function of digital pathways.

### 3.4 Coming together: digital pathways à la carte for people with MS

With regard to the definition of digital patient pathways, categorizing pwMS into different patient groups (subtyping), or the identification of respective patient groups (clustering) in the first place is of particular interest. This is essential to obtain more homogenous patient populations which can be used for defining a set of standard pathways and a common use case for AI/ML.

Once homogenous patient populations have been identified, corresponding pathway prototypes, i.e., standard pathways, can be derived from their data via data and process mining. A prototype pathway equals the chronological sequence of pathway modules belonging to a specified subclass of patients. Designated modules may then be added, deleted or switched to fit the individual patient as needed. Instead of building digital patient pathways from scratch (menu-based), the prototype approach allows practitioners to make intelligent use of any knowledge buried in the data which has been collected from patients in the past (96, 97).

Accordingly, on the premises of a virtual data repository and shared access for all members of the MS care unit, external providers of MS care services and their patients, i.e., all the stakeholders of the pathway, the data stored along the pathway can be processed and analyzed using AI/ML techniques (see also *Section 2.2*).

**Table 4** provides an overview of ML techniques and potential use cases in healthcare in general and MS in particular.

**Table 4:** ML techniques and potential use cases in healthcare/MS care.

ML technique	Use cases under research with regard to healthcare/MS care
**Supervised learning** **(labelled training data required)** a) Classification algorithms b) Regression algorithms ⇨ A known number of classes ⇨ Used for *prediction*	a) Prediction of the diagnostic category for a patient − Subtyping of patients through the identification of specific genetic mutations (genotypes), the cause of disease (endotypes) or the clinical manifestation of symptoms (phenotypes) = *classification* b) Prediction of the degree of functional impairment in a patient [e.g., (107)] − Subtyping patients into phenotypes by modelling motor function decline, disease duration, or the slope of progression = *regression*
**Unsupervised learning** **(no labelled training data required)** ⇨ An unknown number of classes ⇨ Used for *analysis*	a) *Clustering* patients into patient groups [e.g., (108, 109)] − identification of patients who share similar features referring to observable characteristics (phenotyping) or underlying dysfunctional or pathological mechanisms of MS (endotyping) b) Reduction of high-dimensional datasets through the generation of simpler representations of highly complex data = *generalization* − Option 1: find hidden dependencies, drop features that add no or only little information and retain features that add the most important information = *feature selection* – transform the dataset into a lower dimension while keeping important information in order to make it a “good to go” dataset for the further training of other ML algorithms − Option 2: find hidden dependencies, create a new dataset which still contains most of the relevant information by performing a linear or non-linear transformation of the original feature space = *feature extraction*, which then can again be used with other ML algorithms c) Identification of sequences = *association* − Find common patterns and relationships between different features in a dataset – indicate which combinations of features occur most often and which do not − Prediction of disease progression based on the cluster the patient is placed in
**Reinforcement learning** **(no training data needed, but constant feedback is provided to the algorithm)**	Trial and error – the goal is to minimize error a) Drug discovery [e.g., (110)]: virtual generation of the optimal molecules with desired properties; adverse reactions or side-effects are fed back to the algorithm as punishment whereas a improvement in disease course would be fed back as reward b) Digital twin [e.g., (111, 112)]: build a virtual copy of a real patient, let the algorithm sequentially learn as data accrue and provide feedback in order constantly reevaluate the treatment regimen and recommend the best combination of treatment parameter values for keeping the virtual patient as healthy as possible

**Figure 5:**
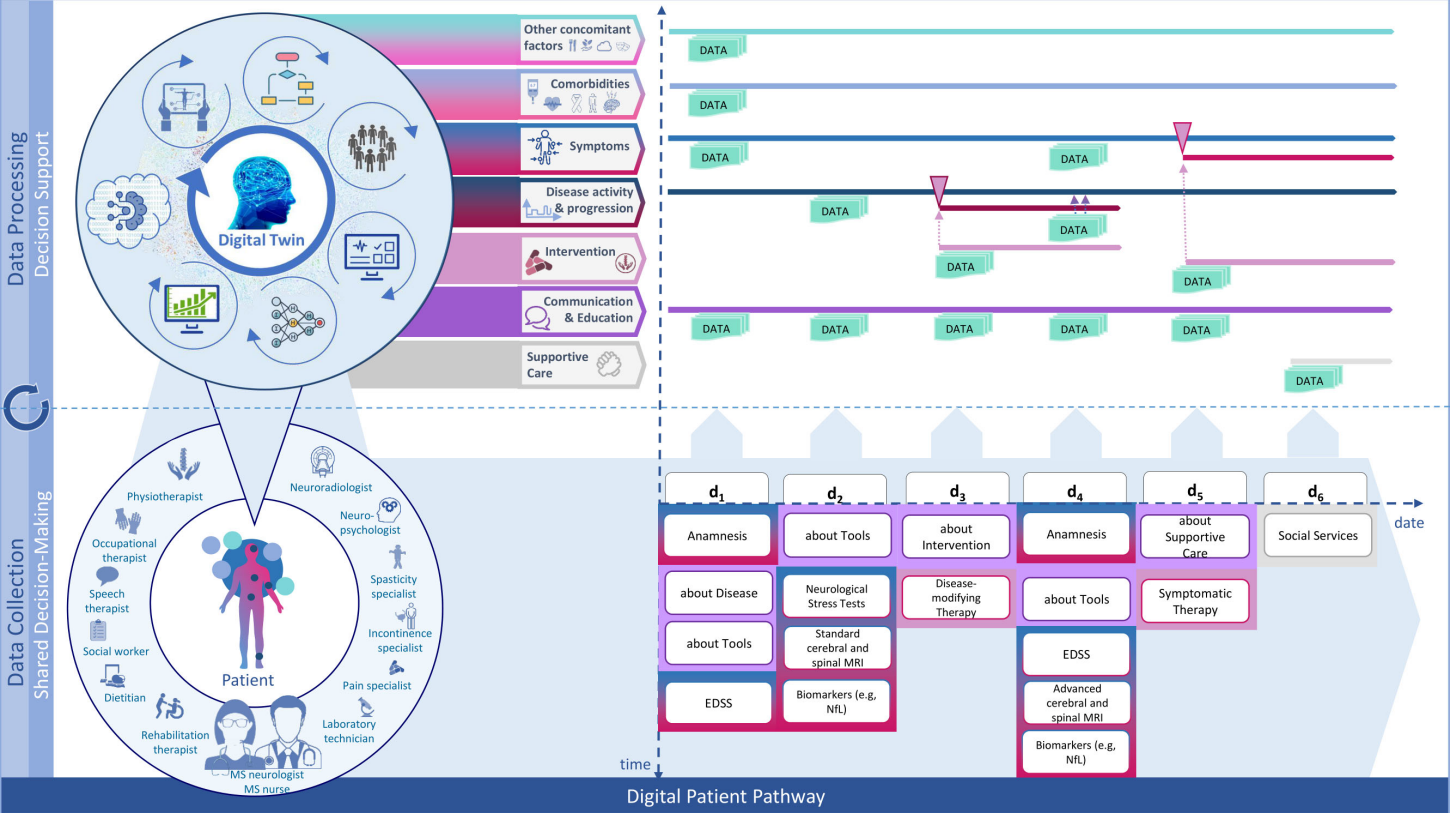
Modular-integrative framework for the design of digital patient pathways. Source: Self-prepared, using icons created by Vecteezy, https://de.vecteezy.com/gratis-vektor.

The journey of the patient can be tracked and modelled along multiple paths as shown in **Figure 5** which allow for a detailed analysis of the individual data that has been collected during the execution of pathway modules. Moreover, this way new individual patient data can be compared to population data fitting the identified subtype of the individual patient. AI/ML-supported variance analysis can then be used to forecast potential future pathways. Based on these, potentially fitting treatment options for the patient may also be identified. In essence, the combination of different ML techniques for these purposes enables the personalization of MS care as it widens the number of dimensions that can be processed to support clinical decision making.

This is how personal digital twins may be generated which mirror different dimensions of accumulated patient data and outcomes. All digital twins of one patient together present the most complete picture of the health state of that particular patient. They add up to being the actual *Digital Twin* of the patient.

In healthcare, clinical decision support systems (CDSS) have already been investigated for a variety of use cases [for a scoping review in the medical field see, e.g., Noll, Schaaf and Storf (113)], e.g., to determine the future direction of disease course and options for therapy (8, 114–116). One prominent example of such a CDSS in MS care is MSProDiscuss (117). In contrast, to the Digital Twin however, existing CDSS do not incorporate data from all dimensions which is why the envisioned Digital Twin would be superior to those.

### 3.5 Walking along the pathway: MS treatment could change for the better

MS symptoms may last for days or weeks but may also disappear again (remission). However, if left untreated most pwMS will develop disease symptoms that will gradually worsen over time (relapsing). At one point in time, there will then be no more discernible relapses and remissions implying that pwMS will have transitioned from relapsing-remitting MS (RRMS) to secondary progressive MS (SPMS) (86).

About 10-15% of pwMS experience gradual worsening from the start, which is thus referred to as primary progressive MS (PPMS). In rare cases, some people with PPMS will also suffer a relapse, which is known as progressive-relapsing MS (PRMS). The majority of patients, however, exhibit RRMS, with percentages of respective total populations studied varying from 76% (76) to 85% (67).

Up until now, the diagnosis of MS implies that pwMS are assigned either one of these phenotypes. With regard to MS, the definition of phenotype traditionally follows the description of the clinical courses of the disease (118, 119), which are also part of the McDonald criteria in their last revised version of 2017. In addition to that, imaging and other paraclinical markers have been incorporated into the criteria over time (6).

In general, the term p*henotype*, however, refers to the observable characteristics of a disease whereas the word *endotype* is used to describe the molecular, pathological or pathophysiological mechanisms underlying a disease (120–122). In the literature, especially with regard to asthma and allergies, it has been highlighted that phenotypes neither necessarily relate to nor provide insights on the underlying pathogenetic mechanisms of a disease. In addition to that, it has been found that phenotypes frequently overlap and may change over time. Therefore, research in the field of asthma and allergies has increasingly been steered towards the identification of disease endotypes, i.e., identifying compilations of disease mechanisms explaining the disease expression in groups of patients (123). In this ongoing discussion, biomarkers constitute a link between both concepts as they may provide information about the pathophysiology of an underlying disease, the course of an illness, and/or the response to treatment (124, 125).

With regard to allergies the argument has been made that the focus on phenotypes where one single phenotype comprises multiple molecular endotypes constitutes an oversimplification diminishing the efficacy of treatment (126). Resolving phenotype populations into endotypes where people with the same endotype exhibit the same disease mechanism has also been advocated in pharmacological research (127, 128) where a paradigm shift towards the targeting of the mechanism(s) and cause of a disease instead of targeting symptoms and affected organs can be observed. This goes hand in hand with a shift towards precision medicine according to which therapies should be tailored to fit the individual patient. Patients within the same endotype are assumed to be more consistent in their response to a selected treatment because of a common pathophysiology (127).

In MS research, it has also been proposed to change the design of clinical trials along these lines: Manouchehri, Zhang et al. (129) have argued that more research into immunopathological factors that drive disease activity is needed. If current clinical and MRI outcome measures were to be replaced by objectively measurable genomic and proteomic biomarkers, trials would likely become more efficient as it would allow us to narrow down the group of patients to be included in a trial in a more targeted manner. This would lead to more homogenous study groups and minimize the number of non-responders therein. Manouchehri, Zhang et al. (129) even go one step further and root their argumentation on the concept of *endophenotypes* which represent a subclass of endotypes: Patients within an endophenotype “share a measurable indicator or pattern of disease that lies along the causal pathway between gene expression and the phenotype” (127).

One promising treatment-sensitive biomarker for disease activity in pwMS currently being studied is the assessment of high-frequency serum neurofilament light chain levels (130). ML and AI techniques are also increasingly employed in the quest for new MS biomarkers [see, e.g., (131)]. One more reason, to finally leave the traditional pathway and to go digital in MS care.

## 4 Implementing digital patient pathways in the real world: challenges to expect

However, getting there will not be easy. The successful implementation of digital patient pathways will greatly depend on patients’ willingness to share their data. Digital patient pathways by design strongly differ from the current implementation of electronic health records as patient would directly benefit from sharing their data along the pathway. In return for their data, they get access to educational contents and instant feedback on their state of health (as far as appropriate) along their pathway. Besides that they are directly connected to a team from a specialized clinic who they can turn to for personal help or by whom they may also be contacted directly if need be. Last but not least, accompanied by their doctor, patients may zoom in and out of their pathway on the virtual dashboard, look at personalized analyses of their data and future prognoses and may thus be able to take a more active stance in managing their disease. From our perspective, all of these things – which we have described in more detail in previous sections – pose a strong incentive for patients to share their data. Even more so in what they stand to gain in terms of drug design and research (see *Section 3.5*).

Despite all these incentives, patients’ willingness to use digital patient pathways may still be hampered by data privacy and security concerns as health data count among the most vulnerable type of data. These kind of concerns, however, do not only apply to the implementation of digital patient pathways, but to the utilization of data and modern technologies in the realm of healthcare in general. E.g., Paul, Maglaras et al. (132) provide a detailed overview of concerns raised in this regard and also describe a variety of safeguard measures that are currently being discussed in the literature and may be applied to reduce the risk of data theft and misuse.

Related to these concerns is the demand for explainability of AI models, which in turn also needs to be considered when AI technology is to be used for pathway modelling, or for the integration of a chatbot or other AI supported functions into digital patient pathways. Stakeholders interested in implementing digital patient pathways ought to keep that demand in mind, which is also already widely being discussed by researchers [see, e.g., (133–135)].

Another major, but also well-studied problem [see. e.g., (136–138)] which might complicate the implementation of digital patient pathways is the lack of interoperability between different information systems and digital applications in the domain of healthcare.

What these challenges will look like in detail and what other hurdles one might have to take will largely depend on conditions prevailing at the time and location of implementation. Suitable strategies for overcoming these challenges therefore will have to be developed as part of the implementation process. But, as indicated, at least the most prominent concerns are also topics of great interest for other researchers and practitioners in the realm of healthcare and there is already a great amount of ideas available that might prove helpful in the process.

## 5 Discussion and conclusion

Ongoing digital transformation processes around the world and across industries have prompted the use of computer-based systems. The development of CDSSs that exploit the full potential of current AI technology and ultimately *Digital Twins*, however, requires agile, interoperable data such that data points, i.e., the multidimensional records from different datasets, as well as individual features thereof can be processed and exchanged separately and in conjunction for the purpose of analyses (139).

In Germany, current implementations of EHRs, information systems and applications used in in- and outpatient settings still lack interoperability, albeit its importance has been widely voiced and acknowledged by different stakeholders (140–143). Developers of CDSSs still likewise fail to adhere to interoperability standards which inhibits their wider adoption in healthcare settings (144).

Once these and other issues (see *Section 4*) have been resolved and digital pathways for MS have been implemented, however, physicians could employ AI technologies to analyze all available health data of a specific MS patient to get a better picture of the individual disease state of that patient (micro level), leveraging recent research findings and group data from MS registries. Researchers, on the other hand, could use AI technologies to analyze combined data sets from multiple MS patients (macro level) to detect any unknown patterns therein, which eventually could lead to the discovery of new endophenotypes and/or endotypes. E.g., Soenksen, Ma et al. (89) have already developed a methodology for the integration of multimodal datasets into AI/ML systems which could serve as a blueprint for future MS research looking to leverage the AI and ML techniques.

It can be concluded that recent advancements in AI technologies could (1) support the personalization of MS care; (2) help us gain new insights in the origin, potential causes and disease triggers of MS; and (3) result in the discovery of new options for therapy. Further research into respective AI applications for the use case MS considering the whole range of multimodal MS data, however, is still needed. Last but not least the implementation of digital pathways has the potential to give us good push heading into this direction. Hence, we consider the modular-integrative framework presented in this paper as a tool for paving the way towards building digital twins for pwMS. Next, we plan to review its usefulness in practice.

## Author contributions

JW: Conceptualization, Project administration, Visualization, Writing – original draft, Writing – review & editing. IV: Conceptualization, Funding acquisition, Writing – review & editing. HI: Funding acquisition, Writing – review & editing. HS: Visualization, Writing – review & editing. TZ: Conceptualization, Funding acquisition, Supervision, Writing – review & editing.
